# Supplementary material for: Radioactive Iodine Treatment and the Risk of Long-Term Cardiovascular Morbidity and Mortality in Thyroid Cancer Patients: A Nationwide Cohort Study
Source: J Clin Med. 2021 Sep 6;10(17):4032. doi: 10.3390/jcm10174032 (PMC8432460; doi:10.3390/jcm10174032)
Supplement: Supplementary file 1 [file jcm-10-04032-s001.zip › jcm-1335169-supplementary.pdf]

Table S1. Characteristics of the control and study groups at the end of study.

Variables		RAI group		Non-RAI group		*P*
		n	%	n	%	
	**Total**	11,889	89.3	1,421	10.7	
**CVD**	Without	10,485	88.2	1,257	88.5	0.40
	With	1,404	11.8	164	11.5	
**CVD-specific mortality**	Without	11,690	98.2	1,393	98.0	0.32
	With	209	1.8	28	2.0	
**Gender**	Male	2,259	19.0	289	20.3	0.12
	Female	9,630	81.0	1,132	79.7	
**Age (years)**		50.0 ± 14.5		49.7 ± 14.4		0.39
**Age groups (yrs)**	20-39	3,220	27.1	390	27.5	0.84
	40-59	5,895	49.6	715	50.3	
	60-79	2,392	20.1	272	19.1	
	≧80	382	3.2	44	3.1	
**DM**	Without	10,923	91.9	1,330	93.6	0.01
	With	966	8.1	91	6.4	
**CKD**	Without	11,678	98.2	1,401	98.6	0.19
	With	211	1.8	20	1.4	
**Hyperlipidemia**	Without	11,614	97.7	1,396	98.2	0.11
	With	275	2.3	25	1.8	
**CCI_R**		0.13 ± 0.39		0.10 ± 0.34		0.002

P: Chi-square / Fisher exact test on category variables and t-test on continue variables

RAI=radioactive iodine; CVD: cardiovascular disease; DM=diabetes mellitus; CKD=chronic kidney disease; CCI_R=Charlson comorbidity index after removal of the above mentioned comorbidities and myocardial injury, chronic heart failure, peripheral vascular diseases, cerebrovascular diseases or transient ischemic attack and hemiplegia

Table S2. Detail numbers in each year of Kaplan-Meier plots for the cumulative risk of CVD and cumulative survival of CVD-specific mortality stratified by radioiodine (RAI) with log-rank test.

**In the tracking of x year(s)**	**Numbers of CVD**			**Numbers of CVD-specific mortality**		
	**With RAI** (n = 11,889)	**Without RAI** (n = 1,421)	**Log-rank** ***P***	**With RAI** (n = 11,889)	**Without RAI** (n = 1,421)	**Log-rank** ***P***
**1**	299	35	0.620	27	1	0.215
**2**	556	62	0.387	63	9	0.800
**3**	779	93	0.515	88	14	0.369
**4**	945	115	0.531	108	19	0.156
**5**	1,060	123	0.462	132	20	0.163
**6**	1,140	130	0.654	152	20	0.507
**7**	1,202	142	0.410	158	22	0.333
**8**	1,241	143	0.457	162	22	0.371
**9**	1,285	146	0.576	172	23	0.391
**10**	1,317	151	0.599	182	27	0.166
**11**	1,344	154	0.817	188	27	0.275
**12**	1,370	157	0.943	196	28	0.281
**13**	1,386	159	0.819	202	28	0.473
**14**	1,398	161	0.748	206	28	0.544
**15**	1,403	164	0.586	209	28	0.657
**16**	1,404	164	0.716	209	28	0.623

RAI=radioactive iodine; CVD=cardiovascular disease;

Table S3. Factors of CVD stratified by variables listed in the table by using Cox regression

	RAI group			Non-RAI group			RAI *vs.* non-RAI *(Reference)*			
Stratified	Events	PYs	Rate	Events	PYs	Rate	Ratio	Adjusted HR	95% CI	*P*
**Total**	1,404	22899.15	6,131.23	164	2744.86	5,974.81	1.03	0.99	0.84-1.16	0.88
**Gender**										
Male	354	4,193.42	8,441.80	45	533.95	8,427.68	1.00	0.92	0.67-1.26	0.59
Female	1,050	18,705.73	5,613.25	119	2210.9	5,382.42	1.04	1.02	0.84-1.23	0.87
**Age groups (yrs)**										
20-39	125	4,476.06	2,792.64	14	556.83	2,514.23	1.11	1.18	0.67-2.08	0.57
40-59	484	10,274.88	4,710.52	54	1246.86	4,330.86	1.09	1.08	0.81-1.43	0.61
60-79	589	6,787.74	8,677.41	72	774.719	9,293.69	0.93	0.90	0.71-1.16	0.43
≧80	206	1,360.47	15,141.79	24	166.443	14,419.33	1.05	1.03	0.66-1.60	0.89
**DM**										
Without	1,138	20,145.62	5,648.87	138	2477.31	5,570.56	1.01	0.98	0.82-1.17	0.83
With	266	2,753.53	9,660.32	26	267.549	9,717.85	0.99	1.00	0.66-1.52	1.00
**CKD**										
Without	1,306	22,315.13	5,852.53	156	2675.02	5,831.73	1.00	0.97	0.82-1.15	0.73
With	98	584.02	16,780.33	8	69.8361	11,455.40	1.46	1.90	0.79-4.60	0.15
**Hyperlipidemia**										
Without	1,321	22,075.17	5,984.10	157	2690.23	5,835.92	1.03	1.00	0.84-1.18	0.96
With	83	823.97	10,073.13	7	54.62	12,815.07	0.79	0.81	0.33-1.97	0.64
**Year**										
2000-2008	845	13,875.12	6,090.04	84	1,526.21	5,503.81	1.11	1.06	0.90-1.26	0.80
2009-2015	559	9,024.02	6,194.58	80	1,218.64	6,564.68	0.94	0.91	0.77-1.07	0.89

P: Chi-square / Fisher exact test on category variables and t-test on continue variables

RAI=radioactive iodine; CVD=cardiovascular disease; DM=diabetes mellitus; CKD=chronic kidney disease;

PYs=person-years; Rate=per 100000 person-years; Adjusted Hazard ratio=adjusted for the variables listed in Table 2; HR=hazard ratio; CI=confidence interval;

Table S4. Factors of CVD-specific mortality stratified by variables listed in the table by using Cox regression

	RAI group			Non-RAI group			RAI *vs.* non-RAI *(Reference)*			
**Stratified**	**Events**	**PYs**	**Rate**	**Events**	**PYs**	**Rate**	**Ratio**	**Adjusted HR**	**95% CI**	***P***
**Total**	209	25642.17	815.06	28	3058.93	915.35	0.89	0.92	0.62-1.37	0.68
**Gender**										
Male	56	4844.64	1155.92	8	608.81	1314.03	0.88	0.80	0.37-1.70	0.56
Female	153	20797.53	735.66	20	2450.12	816.29	0.90	0.93	0.58-1.50	0.78
**Age groups (yrs)**										
20-39	6	4654.19	128.92	0	580.94	0.00	∞	∞	-	0.96
40-59	32	11180.73	286.21	5	1341.64	372.68	0.77	0.82	0.31-2.14	0.68
60-79	94	8039.29	1169.26	14	910.42	1537.75	0.76	0.78	0.44-1.38	0.39
≧80	77	1767.96	4355.30	9	225.93	3983.55	1.09	1.36	0.67-2.79	0.40
**DM**										
Without	179	22280.04	803.41	25	2725.61	917.23	0.88	0.85	0.56-1.30	0.46
With	30	3362.13	892.29	3	333.32	900.03	0.99	1.14	0.28-4.66	0.85
**CKD**										
Without	171	24890.79	687.00	25	2980.98	838.65	0.82	0.87	0.57-1.33	0.51
With	38	751.38	5057.35	3	77.95	3848.64	1.31	2.10	0.47-9.44	0.33
**Hyperlipidemia**										
Without	208	24703.86	841.97	28	2998.96	933.66	0.90	0.92	0.62-1.37	0.68
With	1	938.32	106.57	0	59.97	0.00	∞	∞	-	0.98
**Year**										
2000-2008	121	14,975.25	808.00	14	1,698.29	824.36	0.98	1.01	0.68-1.51	0.61
2009-2015	88	10,666.92	824.98	14	1,360.65	1,028.92	0.80	0.83	0.55-1.23	0.70

P: Chi-square / Fisher exact test on category variables and t-test on continue variables

RAI=radioactive iodine; CVD=cardiovascular disease; DM=diabetes mellitus; CKD=chronic kidney disease;

PYs=person-years; Rate=per 100000 person-years; Adjusted Hazard ratio=adjusted for the variables listed in Table 2; HR=hazard ratio; CI=confidence interval;
